# Stool DNA test targeting methylated syndecan-2 (SDC2) as a noninvasive screening method for colorectal cancer

**DOI:** 10.1042/BSR20201930

**Published:** 2021-01-14

**Authors:** Wei-Chih Su, Wei-Yu Kao, Tsung-Kun Chang, Hsiang-Lin Tsai, Ching-Wen Huang, Yen-Cheng Chen, Ching-Chun Li, Yi-Chien Hsieh, Hsing-Jung Yeh, Chun-Chao Chang, Jaw-Yuan Wang

**Affiliations:** 1 Division of Colorectal Surgery, Department of Surgery, Kaohsiung Medical University Hospital, Kaohsiung Medical University, Kaohsiung, Taiwan; 2 Graduate Institute of Clinical Medicine, College of Medicine, Kaohsiung Medical University, Kaohsiung, Taiwan; 3 Division of Gastroenterology and Hepatology, Department of Internal Medicine, Taipei Medical University Hospital, Taipei, Taiwan; 4 Division of Gastroenterology and Hepatology, Department of Internal Medicine, School of Medicine, College of Medicine, Taipei Medical University, Taipei, Taiwan; 5 Department of Surgery, Faculty of Medicine, College of Medicine, Kaohsiung Medical University, Kaohsiung, Taiwan; 6 Graduate Institute of Medicine, College of Medicine, Kaohsiung Medical University, Kaohsiung, Taiwan; 7 Center for Cancer Research, Kaohsiung Medical University, Kaohsiung, Taiwan; 8 Cohort Research Center, Kaohsiung Medical University, Kaohsiung, Taiwan

**Keywords:** Colorectal cancer, noninvasive, stool DNA test

## Abstract

Despite the steadily increasing worldwide incidence of colorectal cancer (CRC), an effective noninvasive approach for early detection of CRC is still under investigation. The guaiac-based fecal occult blood test (FOBT) and fecal immunochemical test (FIT) have gained popularity as noninvasive CRC screening tests owing to their convenience and relatively low costs. However, the FOBT and FIT have limited sensitivity and specificity. To develop a noninvasive tool for the detection of CRC, we investigated the sensitivity, specificity, and accuracy of a stool DNA test targeting methylated syndecan-2 (SDC2), which is frequently methylated in patients with CRC. The present study enrolled 62 patients diagnosed as having stage 0-IV CRC and 76 healthy participants between July 2018 and June 2019 from two institutions. Approximately 4.5 g of stool sample was collected from each participant for detection of human methylated SDC2 gene. In total, 48 of 62 (77.4%) patients with CRC showed positive results, whereas 67 out of 76 (88.2%) healthy participants showed negative results. The area under the curve of the receiver operating characteristic curve constructed was 0.872 for discrimination between patients with CRC and healthy individuals. The present study highlights the potential of the fecal methylated SDC2 test as a noninvasive detection method for CRC screening with a relatively favorable sensitivity of 77.4%, a specificity of 88.2% and a positive predictive value of 84.2% compared with other available fecal tests. Further multicenter clinical trials comprising subjects of varied ethnicities are required to validate this test for the mass screening of patients with CRC.

## Introduction

Colorectal cancer (CRC) is the third most commonly diagnosed cancer worldwide and the fourth most common cause of cancer-related deaths [1,2]. Several studies have revealed that the mortality rate associated with CRC has steadily decreased over the past two decades owing to early detection through screening to identify and remove adenomatous polyps at the early disease stages, which ultimately increases the survival of patients with colorectal tumors [3–6].

Despite the steadily increasing incidence of CRC worldwide, an effective noninvasive approach for the early detection of CRC is worth further investigation. The guaiac-based fecal occult blood test (FOBT) and fecal immunochemical test (FIT) have gained popularity as noninvasive screening tools for CRC owing to their convenience and relatively low costs [7]. They help to detect human blood hemoglobin (Hb) in a single stool sample [8]. However, the FOBT and FIT have limited sensitivity and specificity, as patients with positive FOBT or FIT results would require colonoscopy to verify the etiology of potential bleeding within the gastrointestinal (GI) tract.

The multitarget stool DNA test is a recent advancement within the past 10 years that incorporated testing for molecular markers of abnormal DNA into the traditional FIT. The sensitivity of multitarget stool DNA test in average-risk adults who underwent colonoscopy was reported to be higher than that of the FIT (92% vs. 74%), despite the test being less specific (87% vs. 95%) than the FIT for CRC [9]. Advanced detection of CRC and effortless diagnosis are essential for cancer prevention and surveillance.

Stool DNA-based DNA methylation assays using several epigenetic biomarkers emerged as a new strategy for identifying patients with both CRC and precancerous lesions [10]. Among the various reported epigenetic biomarkers [11–13], aberrant SDC2 methylation was demonstrated to occur frequently across all stages of CRC through comprehensive methylation analysis of CRC and normal mucosal tissue samples [14]. Several studies have also established that SDC2 methylation can be sensitively and specifically detected in blood and stool samples from patients with CRC [10,15]. Methylated SDC2, in particular, was detected in 159 out of 196 (81.1%) of CRC patients and 71 out of 122 (58.2%) participants with adenomas [15].

To further develop a tool for the noninvasive detection of CRC, in two institutions, we investigated the sensitivity of the stool DNA test targeting methylated SDC2 that is frequently methylated in CRC [15]. The performance of this single-point verification stool DNA test will be validated for evaluation of the cost-effectiveness of this test in routine screening settings.

## Materials and methods

### Patient enrollment

**Figure 1:**
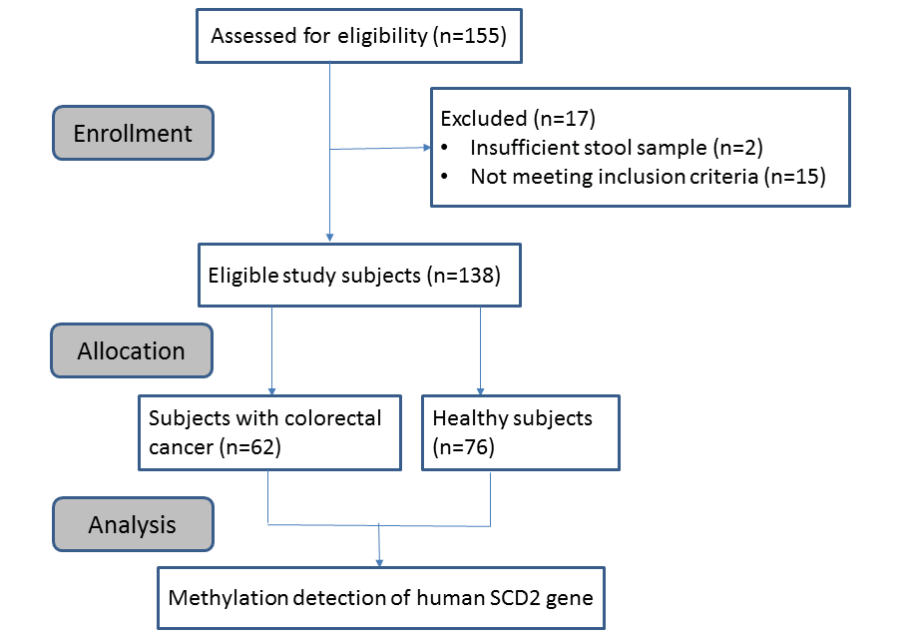
The CONSORT diagram Flowchart of disposition of the study participants.

The present study enrolled 62 patients with CRC who were diagnosed and stage 0-IV CRC and 76 healthy participants between July 2018 and June 2019 from two institutions. Written informed consent was obtained from each participant. The trial was conducted in accordance with the Good Clinical Practice guidelines and the Declaration of Helsinki. The protocol was approved by the Institutional Review Board of Kaohsiung Medical University Hospital (KMUHIRB-G(I)-20180003) and Taipei Medical University Hospital (MU-JIRB-N201805088). The CONSORT diagram is shown in Figure 1.

The demographic characteristics and clinicopathological features of each patient with CRC were recorded. Data including age; sex; histological type; tumor, node, and metastasis (TNM) classification; perineural invasion; vascular invasion; tumor location (distance from anal verge); and tumor regression grade were well documented. The TNM staging was defined according to the criteria of the American Joint Commission on Cancer/Union for International Cancer Control [16]. The TNM system assesses cancer growth and spread in three aspects: the size/extent of the primary tumor (T), absence or presence of regional lymph node involvement (N), and absence or presence of distant metastases (M). Once the T, N, and M categories are determined, a stage of 0, I, II, III, or IV is assigned, with stage 0 being *in situ*, stage I being early, and stage IV being the most advanced disease [16].

### Sample collection

A biodegradable and flushable fecal stool sample collection paper (Feces Catcher; Abbexa Ltd, Cambridge, United Kingdom) with adhesive strips on both sides was stuck onto two sides of the toilet seat to avoid contamination of toilet water, urine, toilet detergents, etc. Approximately 4.5 g of stool sample was collected from each enrolled individual and deposited into a storage tube prefilled with 15 ml of preservative buffer (Creative Biosciences Co. Ltd., Guangzhou, China), using a stool collection syringe within a semiquantitative stool collection device (Creative Biosciences). The resulting buffered stool samples were shipped at 2–8°C to a central laboratory (Preventive Medicine Laboratory of Health GeneTech Corp., Taiwan) and stored at −80°C for methylation detection of human SDC2 gene.

### Methylation detection of human SDC2 gene

A methylation detection kit for human SDC2 gene (Lot# SD21902001; Creative Biosciences) was used to qualitatively detect the methylation of the human Syndecan-2 (SDC2) gene promoter region in the stool samples and determine the risk of CRC in each participant. The tests were performed according to the manufacturer’s protocol, as previously described in detail [15]. Briefly, the test mainly comprised two steps: DNA extraction and transformation as well as fluorescent quantitative polymerase chain reaction (qPCR).

Human DNA was first extracted from the stool sample in which SDC2 gene and β-actin gene were simultaneously captured by a magnetic bead. Stool DNA were purified and enriched with a sequence-specific capture technology as reported before with minor modifications [17]. Each capture reaction was carried out by adding 300 μl of crude stool DNA to an equal volume of 6 mol/l guanidine isothiocyanate solution (Sigma, St. Louis, United States of America) containing two biotinylated sequence specific oligonucleotides (10 pmol total; [15]). After an incubation for 4 h at room temperature, 50 μl prepared Dynabeads® M-280 streptavidin (Thermo, Massachusetts, United States of America) was added to the solution, and incubated for 1 h at room temperature.

The bead/hybrid capture complexes were then washed twice with 1× wash buffer (1.0 M NaCl, 5 mM Tris-HCl [pH 7.5], 0.5 mM EDTA), and then eluted out in 50 μl nuclease-free water with 20 ng/μl transfer RNA (Sigma, St. Louis, United States of America). Target gene SDC2 gene and reference gene β-actin (ACTB) were captured together in a single reaction. Capture probe sequences were listed as previously described [15].

Subsequently, sulfite was used to transform unmethylated DNA, whereas methylated DNA was unaffected. Next, Q-PCR was employed using LightCycler 96 (Roche, Basel, Switzerland) to detect the methylated SDC2 gene and the β-actin gene conserved sequence under the following cycling conditions: 95°C for 5 min; 48 cycles at 95°C for 15 s, 58°C for 30 s, and 72°C for 30 s; and a final cooling step at 40°C for 30 s.

Amplification of methylated SDC2 genes reported by FAM-labeled fluorescent probes was considered as a biomarker for CRC, whereas β-actin amplicons labeled with Texas Red were used as an internal control gene to evaluate the sample DNA. In addition, a positive control (HCT116 cell line DNA with known methylation in SDC2 promoter region) and negative control (Caco2 cell line DNA with no methylation in SDC2 promoter region) were included in every test run. A cycle threshold (*C*t) of qPCR whereby *C*t ≤ 39 denotes a positive stool DNA test result, and *C*t > 39 indicates otherwise [15]. The basis of *C*t 39 cutoff for methylated SDC2 is suggested based on the calculations in Supplementary Table S1. The cutoff is selected to balance the overall sensitivity and specificity (Supplementary Table S1).

### Statistical analysis

Statistical analysis was conducted using Microsoft Excel 2010 and MedCalc (version 18.6; MedCalc Software, Ostend, Belgium; http://www.medcalc.org; 2018). Continuous variables were expressed as medians and IQRs and analyzed using one-way analysis of variance. Categorical variables were analyzed using Fisher’s exact test or the chi-squared test where appropriate. The receiver operating characteristic (ROC) curve was constructed, with area under the ROC curve (AUC) and corresponding 95% confidence intervals (CIs) being calculated for stool DNA test using the R Project for Statistical Computing, Vienna, Austria (http://www.R-project.org/). The cut-off value with the highest accuracy (minimal false-negative and false-positive results) was determined. A *P* value of <0.05 denoted statistical significance.

## Results

### Patient demographics

The demographic characteristics of the 138 enrolled participants are summarized in Table 1. The group of patients with CRC and the healthy group comprised 62 and 76 participants, respectively, with a median (IQR) age of 65 (37–86) and 55 (28–78) years, respectively, and a male-to-female ratio of 1.14 and 0.95, respectively. The left-sided colon (defined as splenic flexure to sigmoid colon) was the most common tumor site (83.9%) in the group of patients with CRC (Table 1).

**Table 1:** Demographics of study subjects (*N*=138)

	CRC subjects (*N*=62)	Healthy subjects (*N*=76)	*P* value
Gender	62	76	
Male	33 (53.2%)	37 (48.7%)	0.596
Female	29 (46.8%)	39 (51.3%)	
Median age (years, range)	65 (37–86)	55 (28–78)	<0.00001
Male	63 (37–86)	53 (28–72)	0.0001
Female	66 (48–81)	57 (39–78)	0.0042
Tumor location			
R't colon/L't colon	10(16.1%)/52(83.9%)	NA	NA
Postoperative pathologic staging			
Tis	8 (12.9%)	NA	NA
I	14 (22.6%)		
II	8 (12.9%)		
III	18 (29.0%)		
IV	5 (8.1%)		

CRC, Colorectal cancer; L't colon, Left-sided colon was defined as splenic flexure to rectum; R't colon, Right-sided colon was defined as cecum to splenic flexure.

### Clinicopathological characteristics and stool DNA test

The clinicopathological variables of all 62 patients with CRC are provided in Table 2. The patients were divided into two groups according to their stool DNA test results: a positive test result group (*C*t ≤ 39; *N*=48) and negative test result group (*C*t > 39; *N*=14). Sex and age did not differ between the positive and negative test result groups (both *P*>0.05). The stool DNA test results were not correlated with tumor sidedness (*P*=0.064, Table 2).

**Table 2:** Clinicopathologic variables of colorectal cancer patients and results of stool DNA test (*N*=62)

	Positive test result (*C*t ≤ 39; *N*=48)	Negative test result (*C*t > 39; *N*=14)	*P* value
Gender			
Male	26 (54.2%)	7 (50%)	0.783
Female	22 (45.8%)	7 (50%)	
Median age (years, range)			0.660
Male	64.5 (37–86)	60 (43–75)	0.569
Female	65.5 (48–81)	68 (50–79)	0.447
Tumor location			
R't colon/L't colon	5(10.4%)/43(89.6%)	5(35.7%)/9(64.3%)	0.064
Depth of tumor invasion			
Tis/T1/T2/T3/T4	6(12.5%)/2(4.2%)/9(18.8%)/21 (43.8%)/1(2.1%)	2(14.3%)/3(21.4%)/2(14.3%)/5 (35.7%)/2(14.3%)	0.166
Lymph node involvement			
N0/N1/N2	24(50.0%)/9(18.8%)/6(12.5%)	8(57.1%)/5(35.7%)/1(7.1%)	0.553
Distant metastasis			
M0/M1	36(75.0%)/3(6.3%)	12(85.7%)/2(14.3%)	0.848
Pathologic tumor stage			
Tis/I/II/III/IV	6(12.5%)/11(22.9%)/6(12.5%)/ 13(27.1%)/3(6.3%)	2(14.3%)/3(21.4%)/2(14.3%)/ 5(35.7%)/2(14.3%)	0.953
Lymphovascular invasion			
Yes/No	4(8.3%)/34(70.8%)	2(14.3%)/12(85.7%)	0.910
Perineural invasion			
Yes/No	4(8.3%)/34(70.8%)	2(14.3%)/12(85.7%)	0.910
Histology			
A/M/N	26(54.2%)/8(16.7%)/0	12(85.7%)/1(7.1%)/1(7.1%)	0.138
Tumor grade			
WD/MD/PD	3(6.3%)/29(60.4%)/2(4.2%)	1(7.1%)/11(78.6%)/0	0.687
Regional lymph node metastases			
Yes/No	15(31.3%)/24(50.0%)	6(42.9%)/8(57.1%)	0.773
Apical lymph node metastases			
Yes/No	1(2.1%)/38(79.2%)	0/14(100%)	0.366
Proximal margin			
Free/Not free	38(79.2%)/1(2.1%)	14(100%)/0	0.366
Distal margin			
Free/Not free	38(79.2%)/1(2.1%)	14(100%)/0	0.366
Circumferential margin (median, range)	2.75 cm (0.1–8.6cm)	5.25 cm (0.1–9.5cm)	0.238

*C*t, cycle threshold of quantitative polymerase chain reaction; A, Adenocarcinoma; L't colon: Left-sided colon was defined as splenic flexure to rectum; M, Mucinous carcinoma; MD, Moderately differentiated; N, Neuroendocrine carcinoma; PD, Poorly differentiated; R't colon, Right-sided colon was defined as cecum to splenic flexure; WD, Well differentiated.

Pathologic tumor depth indicated that most patients in both groups had T3 CRCs. The positive test result group and negative test result group comprised 21 (43.8%) and 5 (35.7%) patients with T3 CRC, respectively; however, no statistically significant differences were observed in pathologic tumor depth, lymph node involvement status, distant metastasis status, and tumor staging between these two groups (all *P*>0.05; Table 2).

Pathologic tumor stage distribution did not differ between the positive test result group and negative test result group (*P*=0.953, Table 2). Adenocarcinoma was the most commonly found histology type, and most tumors were moderately differentiated in both groups. No statistically significant difference was observed in lymphovascular invasion, perineural invasion, proximal margin, distal margin, and circumferential margin between the positive and negative test result groups. The median distance of circumferential margin was 2.75 and 5.25 cm in the positive and negative test result group, respectively (all *P*>0.05, Table 2).

### ROC curve analysis

**Figure 2:**
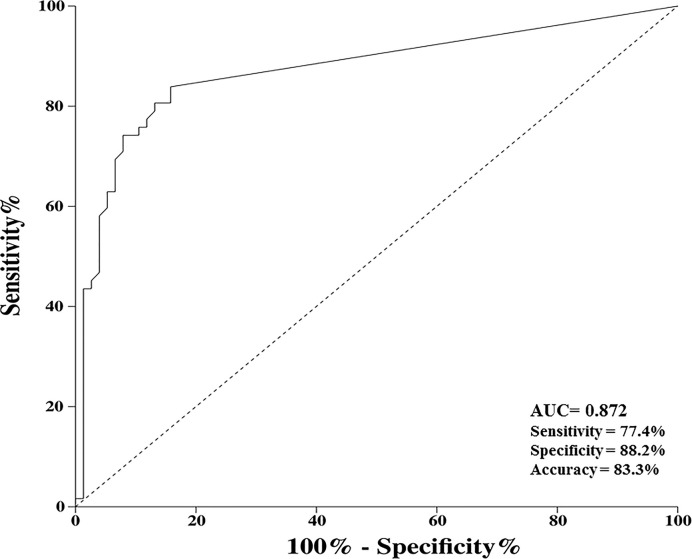
Receiver operating characteristic (ROC) curve of stool DNA tests of all 138 individuals The diagnostic accuracy of detecting methylated SDC2 in stool samples was 83.3% (AUC 0.872, 95% CI = 0.760–0.891), yielding a sensitivity of 77.4% and a specificity of 88.2%.

The sensitivity, specificity, positive predictive value, negative predictive value, and accuracy of the stool DNA test targeting methylated SDC2 are listed in Table 3. ROC curves were constructed using data from the 138 individuals evaluated (Figure 2). Among 62 participants with CRC, 48 showed positive results for the stool DNA test, whereas 67 out of 76 healthy controls had negative results (Table 3). The diagnostic accuracy of detecting methylated SDC2 in stool samples was 83.3% (AUC 0.872, 95% CI = 0.760–0.891), yielding a sensitivity of 77.4% and a specificity of 88.2% (Table 3 and Figure 2).

**Table 3:** The sensitivity, specificity, and accuracy of stool DNA test targeting methylated Syndecan-2 (SDC2)

	Methylation of the Syndecan-2 (SCD2) gene promoter region								
	Colorectal cancer (*N*=62)	Healthy control (*N*=76)	Total (*n*=138)	Sensitivity (95% CI)	Specificity (95% CI)	PPV (95% CI)	NPV (95% CI)	Accuracy (95% CI)	*P*-value
Positive	48	9	57	77.4%	88.2%	84.2%	82.7%	83.3%	<0.001
Negative	14	67	81	(65.0–87.1)	(78.7–94.4)	(72.1–92.5)	(72.7–90.2)	(76.0–89.1)	

95% CI, 95% confidence interval; NPV, negative predictive value; PPV, positive predictive value.

A comparison of performance characteristics of immunochemical, guaiac fecal occult blood tests, a multitarget DNA test, and a stool DNA test targeting methylated SDC2 for detection of advanced colorectal neoplasia is presented in Table 4. A stool DNA test targeting methylated SDC2 in the present study exhibited a distinctively higher sensitivity of 77.4% (95% CI, 65.0–87.1), whereas the sensitivity of various other tests ranged from 2.5% to 46.3% (Table 4). The specificity of immunochemical, guaiac fecal occult blood tests, and multitarget DNA test ranges from 88.3% to 99.9%, whereas the stool DNA test targeting methylated SDC2 has a specificity of 88.2% (95% CI, 78.7–94.4, Table 4).

**Table 4:** Comparison of performance characteristics of immunochemical, guaiac fecal occult blood tests, multitarget DNA test and stool DNA test targeting methylated Syndecan-2 (SDC2) for detection of advanced colorectal neoplasia

	Advanced colorectal neoplasia or colorectal cancer on colonoscopy	Healthy control with no finding	Total subjects (*N*)	Sensitivity (95% CI)	Specificity (95% CI)	Positive predictive value (95% CI)	Negative predictive value (95% CI)
InSure FIT [26]							
Positive	14	30	987	26.3%	96.8%	31.8%	95.9%
Negative	39	904		(15.9–40.7)	(95.5–97.8)	(19.0–47.1)	(94.3–97.0)
OC FIT-CHEK [26]							
Positive	8	20	947	15.1%	97.8%	28.7%	95.1%
Negative	45	874		(6.7–26.1)	(96.6–98.6)	(12.5–46.4)	(93.4–96.4)
Hemoccult II SENSA [26]							
Positive	4	13	1006	7.4%	98.6%	23.6%	94.8%
Negative	51	938		(1.9–17.0)	(97.7–99.2)	(6.3–50.0)	(93.2–96.1)
Hemoccult II SENSA [9]							
Positive	27	7	7904	2.5%	99.9%	79.4%	86.7%
Negative	1046	6824		(1.7–3.7)	(99.8–99.9)	(61.6–90.6)	(85.9–87.4)
HemeSelect [9]							
Positive	22	10	7493	5.0%	99.9%	68.8%	94.4%
Negative	418	7043		(3.2–6.4)	(99.7–99.9)	(49.9–83.3)	(93.8–94.9)
Multitarget DNA test [30]							
Positive	381	732	7104	46.3%	88.3%	34.2%	82.6%
Negative	442	5549		(42.9–49.8)	(87.5–89.1)	(31.5–37.1)	(91.9–93.3)
Stool DNA test for methylated SDC2 ^[current study]^							
Positive	48	9	138	77.4%	88.2%	84.2%	82.7%
Negative	14	67		(65.0–87.1)	(78.7–94.4)	(72.1–92.5)	(72.7–90.2)

InSure FIT, fecal immunochemical test (FIT) that requires testing of two stool samples from different days; OC FIT-CHEK, fecal immunochemical test (FIT) requiring single stool sampling; Hemoccult II SENSA, high-sensitivity guaiac fecal occult-blood test (HS-gFOBT);

HemeSelect, Fecal occult-blood test (FOBT) that identifies human hemoglobin.

## Discussion

The purpose of the present study was to investigate the role of a stool DNA test targeting methylated SDC2 that is frequently methylated in CRC, which is a leading cause of cancer-related morbidity and mortality [18,19], as a potential noninvasive detection tool for the disease. The processes in which normal colonic cells are transformed into premalignant lesions and finally into malignant phenotypes through molecular aberrations have been well studied [20,21]. These molecular aberrations offer an opportunity to screen for CRC and prevent its development [21]. SDC2 is the important candidate target in the present study, as a high frequency of SDC2 methylation was observed in patients with both stage I and later-stage CRC through blood and stool analyses [10,14].

Currently available screening options for CRC are colonoscopy every 5 years or a biennial FOBT using either a high-sensitivity guaiac FOBT (HS-gFOBT) or FIT [22–24]. Colonoscopy, the gold standard for the diagnosis of CRC, has significantly better sensitivity and specificity than the FOBT and FIT for detecting advanced colorectal neoplasia. Owing to its invasive nature, the compliance rate of colonoscopy with or without anesthesia for CRC screening is often lower than that of noninvasive FOBTs and FITs. Many patients still prefer the FOBT or FIT, as colonoscopy has a higher cost and increases the risk of complications [25–30].

The FOBT and FIT have limited sensitivity and specificity, as colonoscopy would eventually be required for patients with positive FOBT or FIT results to verify the etiology of potential bleeding within the GI tract. However, colonoscopy also has a drawback of missing a significant percentage of neoplasms in the proximal colon [31]. A noninvasive approach would be beneficial for cancer prevention and surveillance in mass screening programs for CRC.

Recent advances in the past two decades have focused on novel potential CRC biomarkers, thus enabling stool DNA analysis [32,33]. Three major genetic mechanisms have been reported to be involved in early CRC and precancerous colorectal lesions: (1) chromosomal instability due to mutations in *APC, KRAS*, and *TP53*; (2) microsatellite instability due to a loss of function in mismatch repair genes; and (3) DNA methylation, which is an epigenetic alteration leading to promotor hypermethylation and subsequent suppression of gene transcription [33]. The theory behind stool DNA testing is that advanced benign neoplasms and malignant lesions exfoliate sufficient molecular material to allow for their detection in stool through amplification techniques [34]. Stool DNA could serve as a potentially accurate and noninvasive screening option for CRC.

Established studies have revealed that stool DNA could facilitate the early detection of CRC and advanced adenoma [35]. A significant breakthrough in stool DNA testing was achieved when the multitarget stool DNA test ColoGuard was approved by the FDA of the United States for clinical use based on one multicenter clinical trial that showed that it could detect 42% of adenomas (≥1 cm) and 92% of CRCs, at a specificity of 87% [9]. Although multitarget stool DNA testing detected significantly more cancers than did the FIT in nonsymptomatic patients at an average risk of CRC, it yielded considerably high false positive results [15]. The programmatic sensitivity and specificity of a multitarget stool DNA test (which was previously recommended every 3 years) compared with those of annual FIT screening is unclear. A cost-effectiveness analysis suggested that the FIT is more effective and more economical than a multitarget stool DNA test under most feasible scenarios [36].

Stool DNA methylation of BMP3, NDRG4, SDC2, SFRP2, SEPT9, TFPI2, and VIM had been identified to have diagnostic potential genes for detecting CRC [10,37]. These epigenetic biomarkers had sensitivities ranging from 46% to 90% and specificities ranging from 76.8% to 93.0% [10,33]. Fecal methylated SDC2 demonstrated higher feasibility for detecting colorectal neoplasms in a noninvasive manner, as it identified 159 out of 196 Asian patients with CRC (81.1%) [15]. In total, 71 out of 122 patients (58.2%) who had adenomas were also detected, at a specificity of 93.3% (167/179) [15]. SDC2 methylation is not limited to a single ethnic group; it was also found to be frequently methylated in Australian patients with CRC [38].

Our current study showed that a stool DNA test targeting methylated SDC2 has a relatively favorable sensitivity of 77.4% (95% CI, 65.0–87.1%), a specificity of 88.2% (95% CI, 78.7–94.4%) and a positive predictive value of 84.2% (95% CI, 72.1–92.5) compared with other available fecal tests (Table 4). Commercially available multitarget DNA test was slightly inferior, with a sensitivity of 46.3% (95% CI, 42.9–49.8%) and a positive predictive value of 34.2% (95% CI, 31.5–37.1%) compared with stool DNA test targeting methylated SDC2 in the present study (Table 4).

Unlike the traditional FOBT, a stool DNA test of methylated SDC2 involves no dietary restrictions (including food and medications) that might result in false-positive or false-negative outcomes [39]. No specific dietary restriction is required prior to a stool DNA test of methylated SDC2. It was reported that 13 medicines, animal DNA, plant DNA, and fatty acids did not affect the detection of fecal methylated SDC2 [15]. A stool DNA test of methylated SDC2 is associated with greater patient compliance owing to its noninvasiveness and absence of dietary restrictions.

The present study demonstrated that a stool DNA test targeting methylated SDC2 may be a useful alternative noninvasive screening test for CRC, with a sensitivity of 77.4%. Our finding is in accordance with previous research that supports the feasibility of SDC2 as a methylation biomarker for the detection of colorectal neoplasms [15], and validated its clinical relevance in two Taiwanese institutions. Despite its cost-effectiveness and potential credibility in the clinical screening of CRC, the applicability of a stool DNA test targeting methylated SDC2 requires further research consideration with regard to cost, laboratory accessibility, and qPCR data analysis.

## Conclusion

The present study highlights the potential and scientific viability of a fecal methylated SDC2 test as a plausible alternative noninvasive detection method for screening of CRC. Despite being the first clinical study in Taiwan, the present study has the limitation of having a relatively small sample size; further investigations are warranted on more participants for the mass screening of CRC. Further multicenter clinical trials with international collaborative studies that include other ethnic groups might be required to validate the performance of this stool methylated SDC2 test for CRC screening worldwide.

## Supplementary Material

Supplementary Table S1

## Data Availability

Materials and raw data can be requested from the authors upon request.

## Abbreviations

AUC: area under the curve
CI: confidence interval
CRC: colorectal cancer
Ct: cycle threshold
FIT: fecal immunochemical test
FOBT: fecal-occult blood test
Hb: hemoglobin
qPCR: quantitative polymerase chain reaction
ROC: receiver operating characteristics
SDC2: Syndecan-2
