# Supplementary material for: Stool DNA test targeting methylated syndecan-2 (SDC2) as a noninvasive screening method for colorectal cancer
Source: Biosci Rep. 2021 Jan 14;41(1):BSR20201930. doi: 10.1042/BSR20201930 (PMC7809545; doi:10.1042/BSR20201930)

Supplementary Table S1.

The basis of Ct 39 cutoff for methylated SDC2 is suggested based on the calculations in the following table. The cutoff is selected to balance the overall sensitivity and specificity.

Cutoff CT value	False positive	False Negative	True positive	True Negative	Sensitivity	Specificity	Accuracy
38	6	18	44	70	0.709677419354839	0.921052631578947	0.826086956521739
38.5	8	15	47	68	0.758064516129032	0.894736842105263	0.833333333333333
39	9	14	48	67	0.774193548387097	0.881578947368421	0.833333333333333
39.5	10	13	49	66	0.790322580645161	0.868421052631579	0.833333333333333
40	12	12	50	64	0.806451612903226	0.842105263157895	0.826086956521739
40.5	12	12	50	64	0.806451612903226	0.842105263157895	0.826086956521739
41	12	10	52	64	0.838709677419355	0.842105263157895	0.840579710144927

Supplementary figure 1. The Methylation levels of SDC2 measured in the stools of healthy participants versus CRC patients

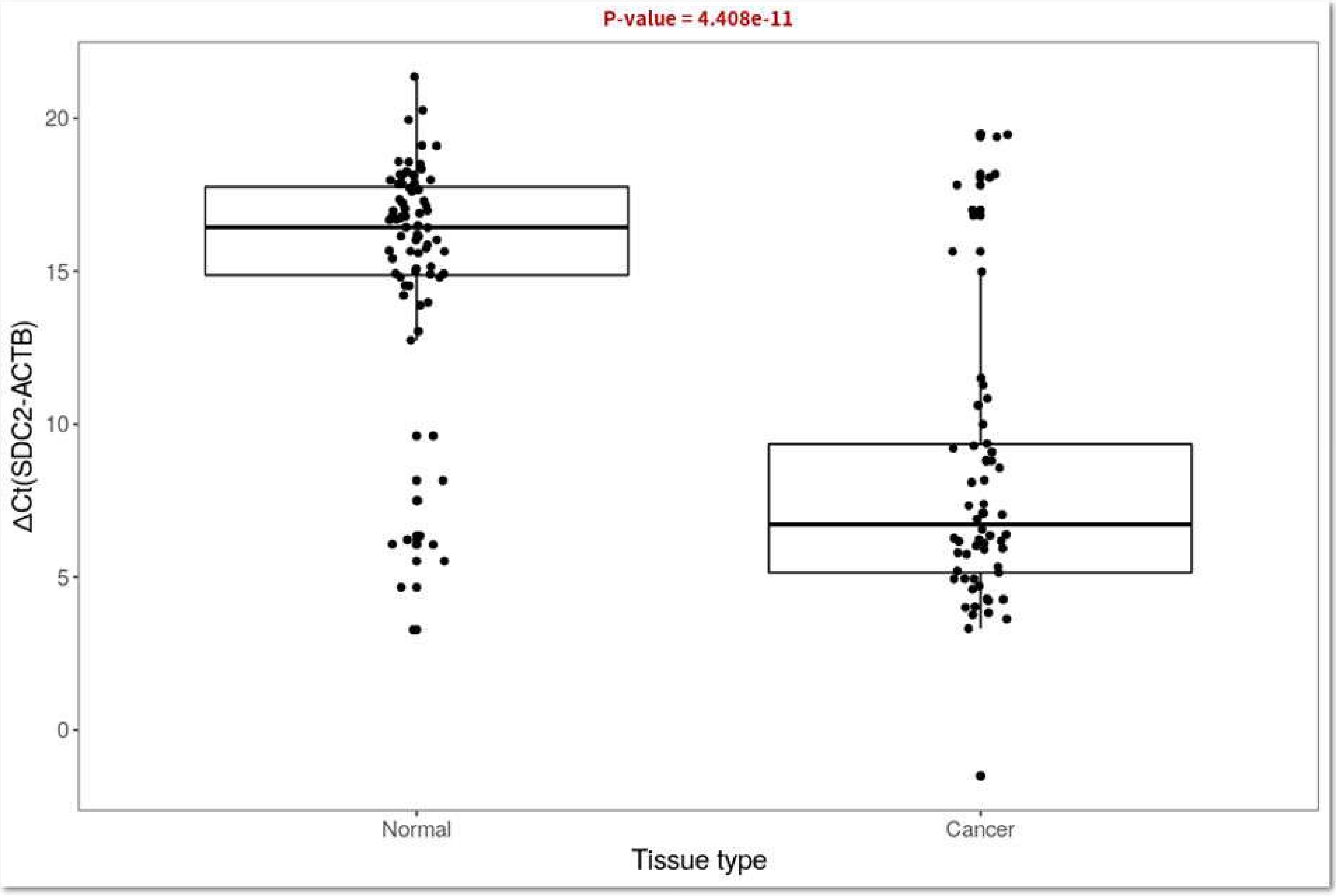

Supplement: Supplementary Table S1 [file BSR-2020-1930_supp.pdf]
